# The Properties of Composites with Recycled Cement Mortar Used as a Supplementary Cementitious Material

**DOI:** 10.3390/ma13010064

**Published:** 2019-12-21

**Authors:** Katarzyna Kalinowska-Wichrowska, Marta Kosior-Kazberuk, Edyta Pawluczuk

**Affiliations:** Faculty of Civil and Environmental Engineering, Bialystok University of Technology, 15-351 Bialystok, Poland; k.kalinowska@pb.edu.pl (K.K.-W.); e.pawluczuk@pb.edu.pl (E.P.)

**Keywords:** recycled cementitious supplementary material, comprehensive concrete recycling, recycled fine fraction, rehydration reactivity

## Abstract

The process of recycling concrete rubble is accompanied by the formation of a large amount of fine fraction, which cannot be reused as aggregate. The results of research on the possibility of using recycled cement mortar (RCM), obtained during concrete recycling, as a cementitious supplementary material, are presented. The experimental research was carried out on the basis of two variables determining the recycling process: *X*_1_—temperature (range of variation 288–712 °C) and *X*_2_—time (range of variation 30–90 min) of thermal treatment of concrete rubble. The experiment included 10 series of new composites made with RCMs subjected to different variants of thermal treatment, and two additional control series. The best treatment parameters were determined based on the assessment of selected physical and mechanical properties of the new cement composites, as well as the analysis of characteristics and microstructure of RCM. The test results showed that proper thermal treatment of concrete rubble makes it possible to obtain a high-quality fine fraction, which has the properties of an active addition and can be used as a partial replacement for cement in mortars and concretes.

## 1. Introduction

Recycling is currently one of the main ways of managing concrete rubble. The huge consumption of concrete in the world and the fact that its manufacturing consumes a large amount of non-renewable natural resources and other materials, e.g., aggregates (80% of concrete mass), Portland cement (10%), supplementary cementitious materials (3%) water (7%), and its production is responsible for 5% of anthropogenic worldwide CO_2_ emissions, encourage a responsible approach to searching for methods and possibilities of its effective recycling [1]. The global aggregate production is currently estimated at 40 billion tons, which is leading to depletion of natural resources and high energy consumption and has negative impact on the environment [2].

The use of recycled aggregates (RA) from construction and demolition waste (CDW) in manufacturing concrete and mortar is a viable way to reduce the unsustainable level of consumption of natural aggregates worldwide and avoid landfilling CDW [3,4].

Research on the recycling of concrete is mainly devoted to finding the most effective way to obtain recycled aggregates of the best quality, which usually means removing the impurities from the surface of the natural aggregate grains. The recycled aggregate’s quality is closely related to the adhered cement paste properties, since the bond between the natural aggregates and the cement paste is usually weak in the interfacial transition zone [5,6,7,8]. It is widely accepted that the presence of cement paste in recycling concrete aggregate causes its worse physical, mechanical and chemical properties compared to natural aggregates. During the process of crushing concrete, even 30–60% of its mass is a fine fraction (<4 mm) containing mainly cement mortar [9]. In order to improve the quality of coarse recycled aggregate, many refining methods (gravity concentration method, heating and rubbing method, mechanical grinding, etc.) have been developed for separating mortar from the surface of its grains [10]. As a result of these treatments, 35% of high-quality coarse aggregate and a total of 65% of the fine fraction containing mainly cement mortar are obtained [11]. An alternative method of treating recycling aggregates was proposed by Tam et al. [12]. They have studied three pre-soaking treatment methods—ReMortarHCl, ReMortarH_2_SO_4_ and ReMortarH_3_PO_4_—aiming at reducing the old cement mortar attached onto the RA. Experimental results show that the water absorption of the pre-treated RA has been significantly reduced with improvement of mechanical properties of the recycled aggregate concrete. A similar approach to construction waste recycling was presented by Robayo-Salazar et al. [13]. The results obtained in their investigation demonstrate the viability of reusing red clay brick waste, concrete waste and glass waste to produce alkali-activated cements that can be used to fabricate blocks, pavers and tiles. The alkaline activators used were solutions of either NaOH or NaOH and water glass.

According to other test results [14,15], the mortar content in recycled aggregates may be as high as 41% of the volume of the concrete rubble. Many studies indicate that this material cannot be used as a fine aggregate, as it significantly worsens the properties of concretes prepared with its use [16]. Therefore, other methods of using it in cement composites should be sought, and such attempts have been made by researchers for several years.

Gastaldi et al. [17] and Schoon et al. [15] used up to 30% of fine recycled material (grain diameter <63 µm) mainly for the production of Portland clinker, obtaining favorable results in terms of C_2_S content in the clinker. Considering the significant amount of non-hydrated cement in the fine fraction of recycled concrete, Bordy et al. [18] presented the results of studies of composite in which part of the cement was replaced with finely ground (to diameter below 80 µm) powder made by crushing and milling of cement paste made in laboratory conditions. It was observed that there was about 24% of active clinker in the cement paste that could be rehydrated. Zhao et al. [16] observed the effect of water saturation of fine fraction from recycled aggregates, used as a partial cement replacement, on the properties of fresh mortar and mechanical properties of hardened new material and the microstructure of interfacial transition zone. Test results showed that mortars made of recycled dry fine aggregate were characterized by higher compressive strength than mortars with saturated aggregate due to the reduced interfacial transition zone.

Some researchers [19,20] studied the rehydration reactivity of fine fraction from recycled concrete after its heating at different temperatures. The results confirm that cement paste heated at a sufficiently high temperature is dehydrated. Particularly as a result of portlandite decomposition, reactive lime is formed. As a result of re-contact with water, it regains the ability to rehydrate. Ahmari et al. [21] proposed the production of a new geopolymeric binder from ground waste concrete powder mixed with fly ash, which can then be used with recycled concrete aggregates to produce new concrete. Tests carried out by some researchers [22,23] have proved that recycled mortar contains non-hydrated cement, calcium hydroxide (CH) and dicalcium silicate (C_2_S), which are capable of hydration and creation of rehydration products. Some research has shown, however, that the mortar remaining on the surface of the recycled aggregates stored outside for a longer time does not show any rehydration reactivity [24]. Nevertheless, a study carried out by Vegas et al. [25] of the mix proportions and characteristics of mortars made with recycled concrete aggregate showed that up to 25% recycled aggregate can be used in cement-based masonry mortars with no significant decline in performance and no new admixtures or higher cement content required. Braga et al. [26] have analyzed the behavior of cement mortars using fine recycled fractions as a substitute for natural sand. In this study, 15% of the required natural sand was replaced by recycled aggregate. An increase in compressive strength with a simultaneous decrease in modulus of elasticity and an increase in water absorbability in comparison to traditional cement mortars were observed.

Considering the need to manage whole concrete rubble, the authors have developed a method for comprehensive recycling of concrete. The method allows obtaining high-quality secondary aggregate and a fine fraction that can be used as a partial cement replacement. Research on the effect of recycled aggregate on new concrete properties has been described in [8].

The aim of the now presented research work was to determine the effect of thermal and mechanical treatment of concrete rubble on the properties of the fine fraction and to assess the possibilities of using waste cement mortar as cementitious supplementary material. For this purpose, two variables have been analyzed: calcination temperature and time of thermal treatment of concrete rubble. The objects of the study were the composites in which part of the Portland cement had been replaced with recycled cement mortar (RCM) after thermal treatment. The RCM applicability as a reactive supplementary cementitious material was assessed based on such composite properties as compressive strength, flexural strength and water absorbability. X-ray diffractometry (XRD), differential thermal analysis (DTA), thermogravimetry analysis (TG) and scanning electron microscopy were used to characterize the microstructure of RCM and also to explain its rehydration reactivity. The conducted research partially resulted in issuance of a patent (PAT 229887 [27]).

## 2. Technology of Recycled Cement Mortar (RCM) Production

### 2.1. The Initial Concrete Rubble

The recycled cement mortar (RCM) was obtained from 2-year-old laboratory cube specimens 100 × 100 × 100 mm due to the recycling process according to PAT 229887 [28]. The composition of the initial concrete mix is presented in Table 1. The concrete was classified as C30/37 strength class.

### 2.2. The Recycling Process of Concrete (Thermo-Mechanical Treatment According to PAT.229887)

**Figure 1:**
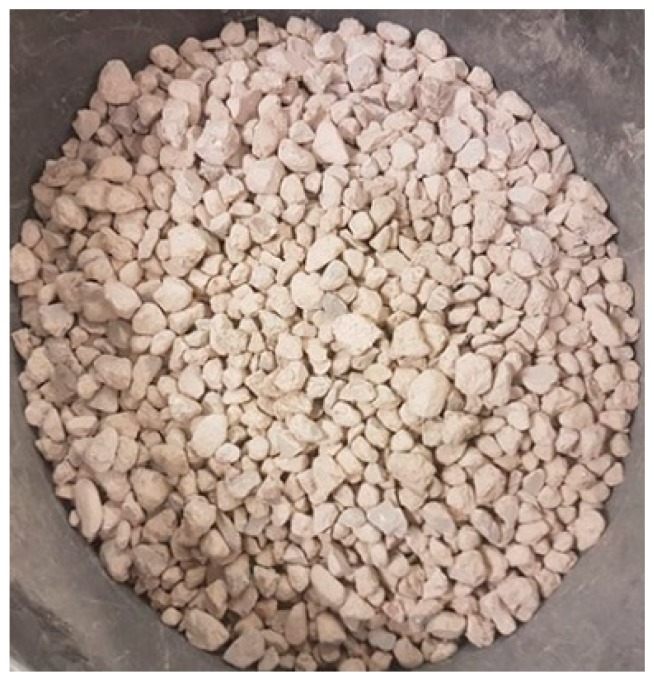
Recycled coarse aggregate obtained after thermal and mechanical treatment.

**Figure 2:**
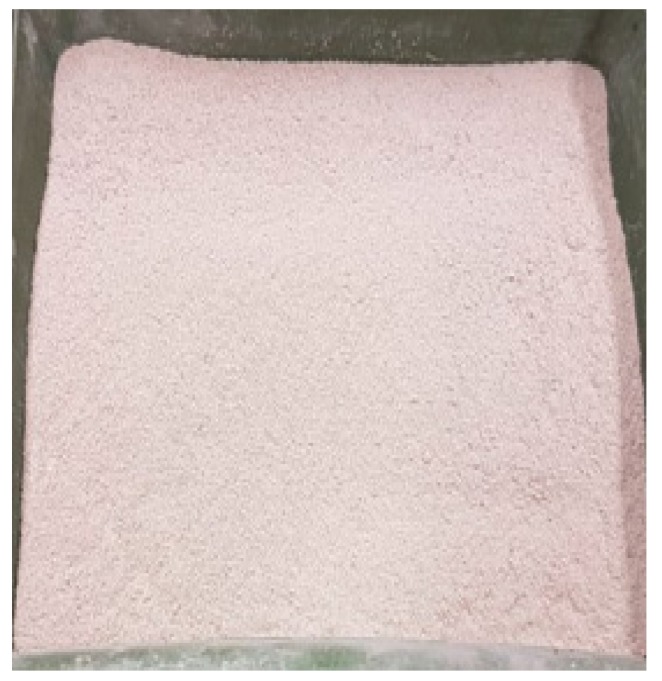
Recycled cement mortar (fine fraction) obtained after thermal and mechanical treatment, used to tests.

The initial crushing of previously disintegrated concrete specimens (100 × 100 × 100 mm) was carried out in a jaw crusher. The concrete was crushed to the grain size *d* ≤ 40 mm. In the next step, the concrete rubble was calcined in a ceramic laboratory furnace. Calcination of the recycled aggregate is necessary in order to dehydrate the cement paste. Dehydration of the cement paste reduces adhesion of the hardened mortar to the aggregate grains and eventually makes it easier to remove it from the surface of the aggregate. After thermal treatment and cooling, the recycled material was mechanically processed in a Los Angeles machine (Merazet, Poland). The grinding time was 15 min. This allowed for the final separation of the recycled cement mortar (RCM) from the surface of coarse aggregate grains. In the last step, the recycled material (fine and coarse fraction) was sieved in order to separate recycled cement mortar (<4 mm) from coarse aggregate (>4 mm). The recycled coarse aggregate and recycled cement mortar after the full process of recycling are shown in Figure 1 and Figure 2. The coarse aggregate (>4 mm) obtained in recycling process can be used in new concrete as a substitute of natural aggregate. The test results of properties of concrete with recycled aggregate were presented in [8].

## 3. Materials and Methods

### 3.1. Materials

**Figure 3:**
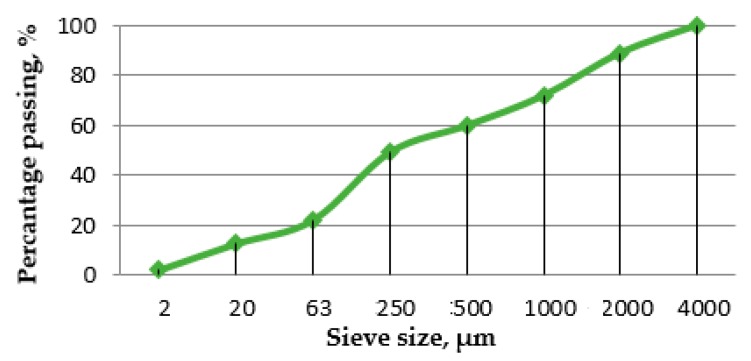
Grading curve of RCM after recycling process.

Portland cement CEM I 42.5 R, meeting the requirements of the EN 197-1:2011 [28] standard, was used for manufacturing cement composites. The natural aggregate sand with a maximum size of 2 mm was used. The procedure of obtaining RCM was described in Section 2.2 and in [23]. For further studies, only the fraction <250 µm of RCM was used as 25% cement replacement. RCM sieve analysis was performed in accordance with EN 933-1:2012 [29], and the grading curve of RCM is presented in Figure 3. As shown in Figure 3, after the recycling process, approximately 20% of the fraction classified as fine aggregate (<4000 µm) according to EN 206:2016 [30] is the dust fraction (<63 µm). For further studies, the fraction <250 µm was used, which was about 50% of the whole material. In future applications, an additional grinding of RCM is recommended immediately after the recycling process to increase the proportion of the finest particles.

### 3.2. The Cement Composites with RCM

The mix compositions of composites with RCM used as a partial cement substitute are shown in Table 2. The compositions were designed as standard cement mortar according to EN 196-1:2016 [31]. In the series 1–10, 25% of the mass of Portland cement was replaced by RCM after thermal and mechanical treatment (RCM calcination temperature and time of treatment were based on the assumptions of the experimental plan, which is described in detail in Section 4). Series 12 had an analogous composition, but the RCM used was not subjected to calcination. Series 11 was made as a standard cement mortar: it did not contain RCM.

### 3.3. Methods

#### 3.3.1. Physical and Mechanical Properties Test Methods

Specimens of composites (40 × 40 × 160 mm) were prepared in accordance with EN 196-1:2016 [31]. After 28 days of curing, the compressive strength and flexural strength tests were carried out [31]. The water absorbability test was performed by determining the percentage increase in the weight of the specimens saturated with water in relation to the weight of the specimen in the dry state. The consistency measurement of fresh cement composite mixtures was made by the flow table method according to EN 1015-3:1999 [32]. The strength activity index (SAI) of the RCM was determined according to EN 450-1:2012 [33]. The heat of hydration of RCM was tested using a semi-adiabatic method based on the standard EN 196-9:2010 [34].

#### 3.3.2. Analysis of RCM Properties and Microstructure

In order to determine the effect of the thermal and mechanical treatment on the phase composition of the RCM, X-ray diffraction analysis was conducted using a D8 Discover A25 instrument (Bruker) with CuKα radiation. All diffraction patterns were obtained by scanning the goniometer from 10 to 70 (2θ) at the rate of 0.05 min^−1^.

The differential thermal analysis and thermogravimetric analysis were carried out using a model STA 409 PG analyzer (Netzsch, Selb, Germany) under a nitrogen atmosphere. The specimens were heated at rate of 10 °C/min to the temperature 1100 °C. The content of RCM components was calculated using DTA/TG DTG curves based on the instructions [35,36].

The morphology of RCM and cement composites with RCM was investigated using a Tescan high-resolution scanning microscope (Aztek Automated, Oxford Instruments, UK) equipped with an X-ray microanalysis system based on the method of X-ray spectrometry with energy dissipation (EDS) and a high-resolution microscope (Quanta 250 FEG, FEI, ThermoFisher Scientific, USA), digitally controlled and equipped with an electron gun with thermal field emission (the Schottky emitter). The shapes of fine particles were classified according to EN ISO 3252:2002 [37].

## 4. Design of the Experiment

### Selection of Variables and Development of the Experimental Plan

For better understanding the relations among the factors determining the characteristics of RCM as a partial substitute for cement, an experiment was performed based on two variables: *X*_1_—temperature of concrete rubble calcination, *X*_2_—time of thermal treatment. The range of variation and the levels of analyzed factors are shown in detail in Table 3.

The calcination temperatures at which the effects of phase changes can be expected were selected. At temperatures up to 350 °C, dehydration of C–S–H silicates, hydrated aluminates and aluminum calcium sulphates occurs along with gypsum decomposition. However, up to 650 °C, portlandite breaks down into CaO and H_2_O. The temperature 500 °C is the center of the plan, and the other extreme like 288 °C and 712 °C are the star points and result from the construction of the adopted rotational plan.

Statistical analysis was carried out in accordance with the rotatable central composite design with a double repetition of the experiment at a central point. The design of the experiment (DoE) enables to check repeatability of results, to find which input factors and their interactions can influence the output properties significantly, to calculate regression equation and to check its adequacy with the test results. The following output properties were selected for analysis: compressive strength, flexural strength and water absorbability of composites with RCM.

On the basis of the above-mentioned variables, the experimental plan including 10 test series and 2 additional control series was established. Table 4 shows the detailed experimental plan with the real and normalized values of the variables.

Apart from the series described in Table 4, additional series were also tested for comparison 11 and 12. For the first control series 11, the cement composite included only cement as a binder, while the other control series 12 was made with RCM without thermal treatment.

Combinations of real values of the examined factors *X*_1_ and *X*_2_ were established on the basis of the assumptions of the design of experiment [38]. The dimensionless normalized values—*x*_1_ and *x*_2_—related to them were used to develop the functions describing the influence of the analyzed factors on the resulting quantities.

The test results were statistically analyzed in order to determine an approximating function describing the influence of the tested variables on the selected properties of the composites with RCM. The analyses included analysis of variance, calculation of regression coefficients and assessment of the regression coefficients’ significance. The function describing the changes in the physical and mechanical properties of cement composites adopted the form of a second-degree polynomial (1):

*y* = *b*_0_ + *b*_1_*x*_1_ + *b*_2_*x*_2_ + *b*_3_*x*_1_*x*_2_ + *b*_4_*x*_1_^2^ + *b*_5_*x*_2_^2^ (1)

where: *y*—dependent variable, explained; *x*_1_, *x*_2_—independent variables; *b_i_*—coefficients; *b*_0_—free term in expression. Calculations were performed according to [38] using software Statistica Version 13 (StatSoft, Poland).

## 5. Test Results and Discussion

### 5.1. Characteristics of Recycled Cement Mortar (RCM)

#### 5.1.1. Heat of Hydration

**Figure 4:**
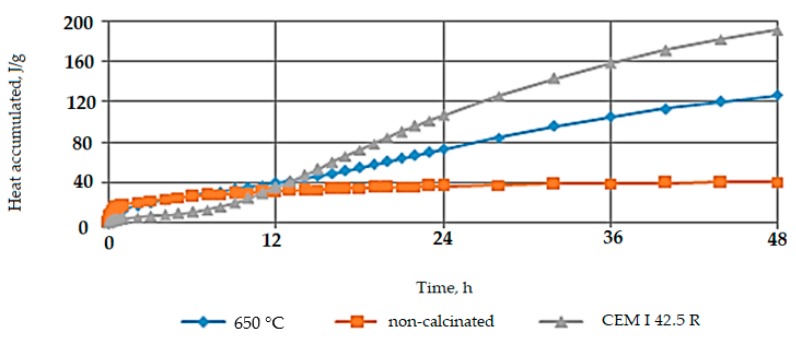
Changes in the amount of heat accumulated of tested materials.

In order to assess the rehydration reactivity of RCM, calorimetric tests were performed, and the results are presented in Figure 4. Two types of RCM were analyzed: calcined at the temperature 650 °C for 60 min and non-calcined material (without thermal treatment). CEM I 42.5 R was used as a standard for comparison. The specific density of RCM equal to 2.66 g/cm^3^ was slightly lower than cement CEM I 42.5 R density, which was 3.05 g/cm^3^.

Based on the analysis of data in Figure 4, it can be concluded that the thermal treatment of RCM has a significant impact on its rehydration reactivity properties and applicability as an active supplementary material. Non-calcined RCM showed no change in the value of heat released over a 48-h measurement period, and the maximum recorded value of heat accumulated was 40 J/g. The RCM calcined at the temperature 650 °C showed the best rehydration reactivity, the amount of accumulated heat increased successively during the test, and after a 48-h measurement period it reached the level of 125 J/g. In relation to the value of accumulated heat obtained for CEM I 42.5 R, it was a decrease of 35%, while compared to non-calcined RCM, an increase of almost 70% was observed.

#### 5.1.2. Strength Activity Index (SAI)

**Figure 5:**
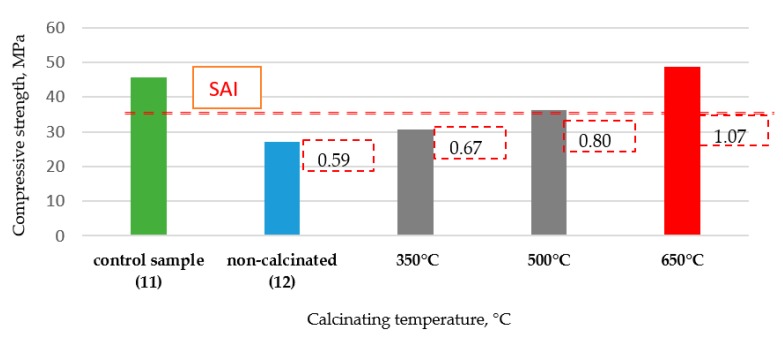
Compressive strength of the composites with and without RCM after 28 days of curing with the strength activity index (SAI).

The RCM’s ability to function as an active addition was assessed on the basis of its pozzolanic properties. The SAI index was calculated according EN 450-1:2012 [33] as mentioned in Section 3.3.1. The SAI of the tested material should be ≥0.75 after 28 days of maturation. Figure 5 presents the strength activity index (SAI) test results for composites with 25% addition of RCM as a cement supplement (compositions according to Table 2) after 28 days of curing for different calcination temperatures and the same time of calcination, which was 60 min. The results were compared to control mortar made of Portland cement (series 11, Table 2).

As seen in Figure 5 the highest compressive strength was obtained for the composite containing 25% of the addition in the form of the RCM calcined at the temperature 650 °C for 60 min (SAI reached the highest level, SAI = 1.07). The increase in SAI value for these samples compared to the non-calcined RCM (series 12, SAI = 0.59) was 79%, and it was 66% compared to RCM calcined in 350 °C (SAI = 0.67). The composites with RCM calcined at temperature 500 °C also confirmed the requirements of standards [34], (SAI = 0.80), but the compressive strength reached 25 and 20% lower than the strength of the composites calcined at 650 °C and the control series, respectively.

#### 5.1.3. X-ray Diffractometry

**Figure 6:**
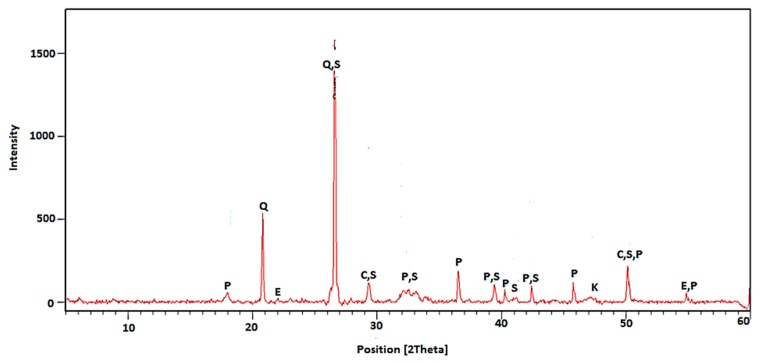
XRD pattern for RCM without thermo-mechanical treatment (Q—quartz, P—portlandite, C—calcium silicate hydrate, S—belite, K—calcite, E—ettringite).

**Figure 7:**
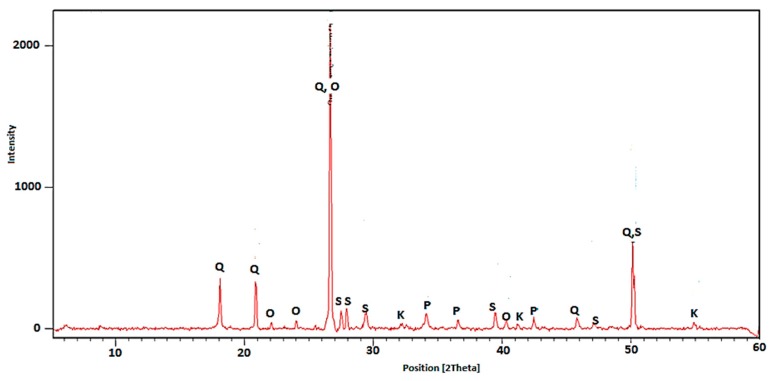
XRD pattern for RCM after thermo-mechanical treatment at temperature 650 °C (Q—quartz, P—portlandite, S—belite, K—calcite, O—calcium oxide).

In order to determine the effect of thermal treatment on the phase composition of RCM, the X-ray diffractometry test was performed. The X-ray patterns for non-calcined RCM and thermally treated specimen are shown in Figure 6 and Figure 7, respectively.

The main crystalline phases found in RCM without thermo-mechanical treatment (Figure 6) are C–S–H gel, belite (C_2_S), portlandite (CH), ettringite, and calcite (CaCO_3_). The XRD pattern of the RCM after thermo-mechanical treatment indicates that some diffraction peaks of the preheated samples gradually disappear, such as ettringite and C–S–H gel. When the heating temperature is raised to 650 °C, it is mainly composed of belite (C_2_S), CaO, partial CH and non-crystalline dehydrated phases.

As expected, in case of material not subjected to the calcination process (Figure 6) the peaks associated with the presence of portlandite were quite intensive and frequent. In case of RCM after thermal treatment, only a single peak was observed. This indicated a properly selected treatment temperature, allowing for almost complete Ca(OH)_2_ decomposition. The small peaks from portlandite in the specimen subjected to calcination (Figure 7) can be explained by high hygroscopicity of disintegrated RCM. The finely ground and calcined RCM contains active CaO, which may react with moisture contained in the air. This phenomenon could not be avoided during sample preparation for XRD, hence the presence of a secondary portlandite in phase composition. Moreover, the calcined sample revealed higher and more frequent peaks, indicating the presence of Portland clinker components (belite), which are responsible for reactivity with water and for the hydration process. This could explain the applicability of calcined RCM as a pozzolanic additive and active filler [35,36]. The above-mentioned components of RCM facilitate further hydration and reaction with new cement paste, which cause the improvement of physical properties of cement composites. This is confirmed by the observations of other authors, who have noticed the content of non-hydrated cement, calcium hydroxide (CH), and dicalcium silicate (C_2_S) in RCM, which are capable of hydration and creation of rehydration products [19,20,22].

#### 5.1.4. Thermogravimetry (TG) and Differential Thermal Analysis (DTA)

**Figure 8:**
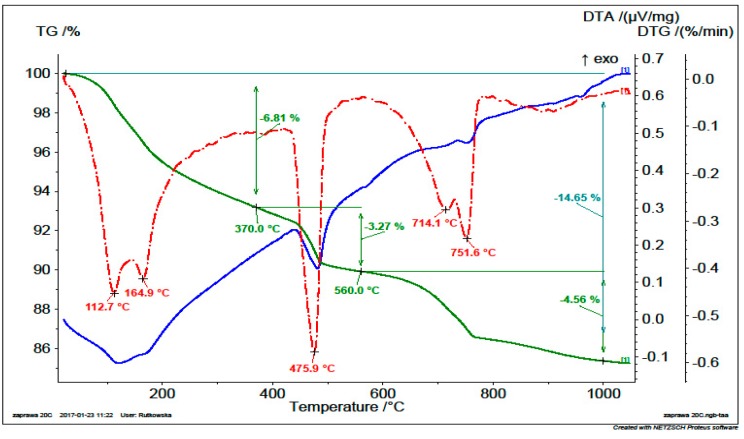
Thermal changes of RCM without thermal and mechanical treatment.

**Figure 9:**
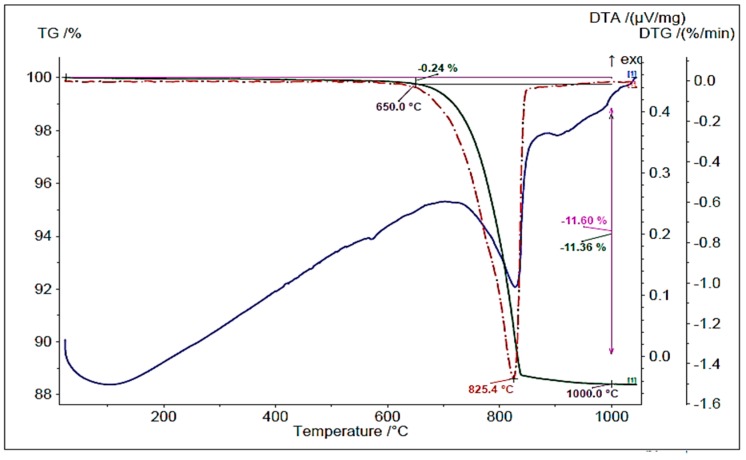
Thermal changes of RCM after thermal and mechanical treatment at temperature 650 °C.

In order to determine the effect of thermal treatment on the content of calcium hydroxide and calcium carbonate in RCM, the samples were subjected to thermogravimetry and differential thermal analysis. In Figure 8 and Figure 9, the weight losses of material when heated to 1100 °C are presented for non-calcined and calcined RCM (at a temperature of 650 °C), respectively.

Table 5 presents the content of bound water, portlandite and calcite in RCM specimens calculated on the basis of plots in Figure 8 and Figure 9 and according to [35,36].

The quite high content of calcium carbonates in both tested specimens is noteworthy. It results from the applied heating temperature equal to 650 °C, which does not cause the decomposition of CaCO_3_. This phenomenon occurs at a temperature above 750 °C, as indicated by peaks associated with mass losses in Figure 8 and Figure 9. The higher CaCO_3_ content in calcined RCM can be explained by the presence of aggregate in the tested specimen. In the RCM specimen after heat treatment, however, there was no peak of portlandite, which confirmed a sufficiently well-selected RCM treatment temperature (Figure 9). Earlier heating of concrete rubble resulted in the disintegration of calcium hydroxide into calcium oxide and water, as evidenced by its lack in the tested RCM specimen in comparison with the untreated specimen. In the presence of water, free calcium oxide has the ability to undergo rehydration, as evidenced by the test results obtained.

#### 5.1.5. SEM Images Analysis

**Figure 10:**
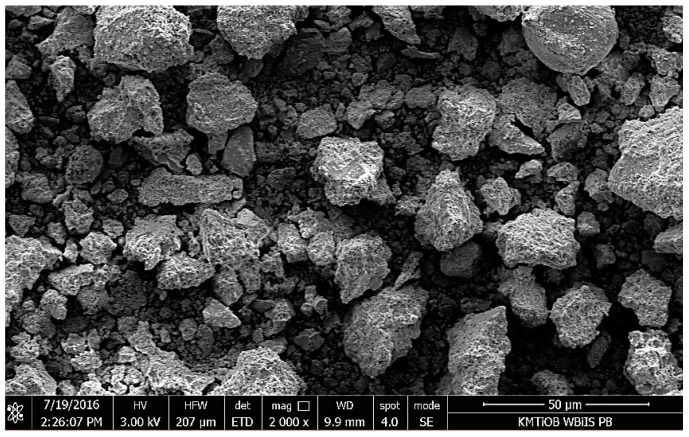
SEM micrograph of RCM particles, mag. 2000×.

**Figure 11:**
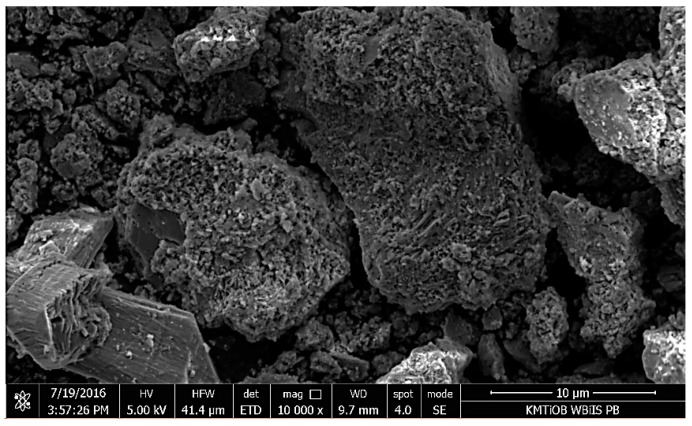
SEM micrograph of RCM particles, mag. 10,000×.

**Figure 12:**
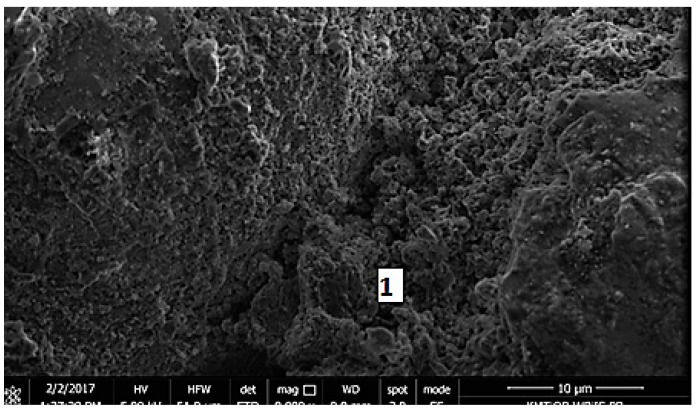
SEM micrograph of cement composite with RCM addition, 1—C-S-H gel (mag.10,000×).

**Figure 13:**
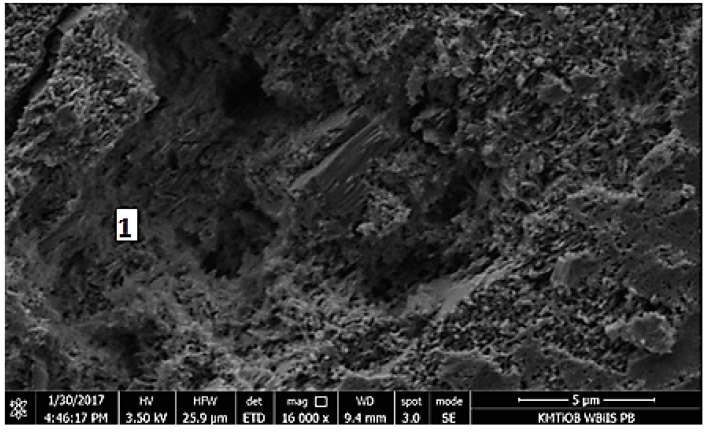
SEM micrograph of cement composite with RCM, 1—C-S-H gel (mag. 16,000×).

**Figure 14:**
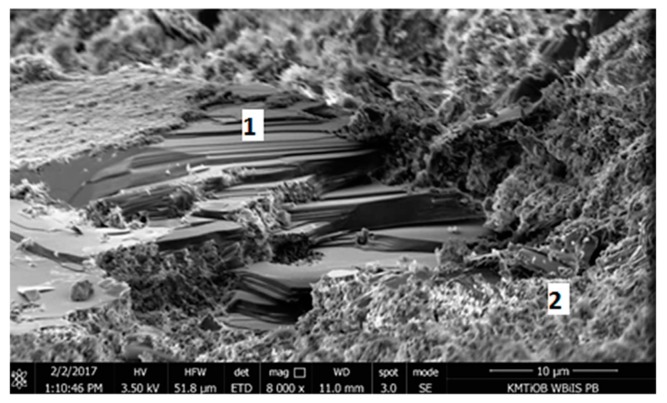
SEM micrograph of microstructure of composite with RCM after thermal and mechanical treatment, 1—portlandite, 2—C-S-H gel (mag. 8000×).

Micrographs of RCM are shown in Figure 10, Figure 11, Figure 12, Figure 13 and Figure 14. The shape of RCM particles (Figure 10 and Figure 11) was defined as irregular polyhedral [37], and they were very similar to cement grains. On the surface of the RCM particles, crystalline inclusions have also been observed, which may probably be the remains of hydrates from the primary hardening process, e.g., parts of non-hydrated cement, free calcium, as well as calcium silicates, as indicated by the results of the phase composition test presented in Section 5.1.3.

Micrographs of the microstructure of cement composites with RCM are presented in Figure 12 and Figure 13. Observations of the microstructure of cement composites (Figure 12 and Figure 13) confirmed that RCM can be perfectly embedded in the cement matrix, forming a compact microstructure. The compact C-S-H phase can be observed in Figure 12 and Figure 13. The compact microstructure may result from strong bonding of high-quality recycling mortar containing C-S-H phases to the components of new paste, as well as the developed surface of the recycled fraction due to applied thermal and mechanical treatment [20].

As it has been noted, the rough surface of the aggregate (in this case also RCM particles) deteriorates the portlandite orientation [39]. The arrangement of portlandite plates (CH) and the C-S-H phase surrounding them are presented in Figure 14.

The orientation of portlandite crystals is additionally disturbed by the presence of Ca(OH)_2_, which, being the nuclei of crystallization of this phase, causes the growth of calcium hydroxide crystals in various directions, improving mortar strength properties [39].

### 5.2. Properties of Fresh Mixtures

Selected test results of the consistency of cement composite mixtures measured according to [28] are presented in Table 6.

The fresh mortar with RCM obtained as a result of the thermal and mechanical treatment (650 °C, 60 min) of concrete rubble was characterized by slightly limited flow diameter in comparison to control mortar. The RCM, due to the developed specific surface (about 3000 cm^2^/g), was also characterized by high water demand. Despite this, it was not necessary to increase the amount of water in the recipe in order to thoroughly mix the ingredients and achieve the required workability.

### 5.3. Properties of Hardened Cement Composites

#### 5.3.1. Compressive Strength

**Figure 15:**
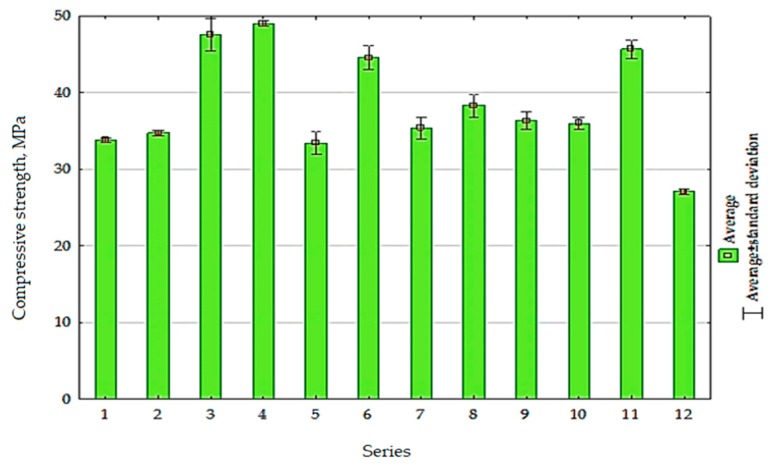
The compressive strength of composites after 28 days.

The average compressive strength results obtained for the test series in comparison to the control samples (series 11) and (series 12) are given in Figure 15.

**Figure 16:**
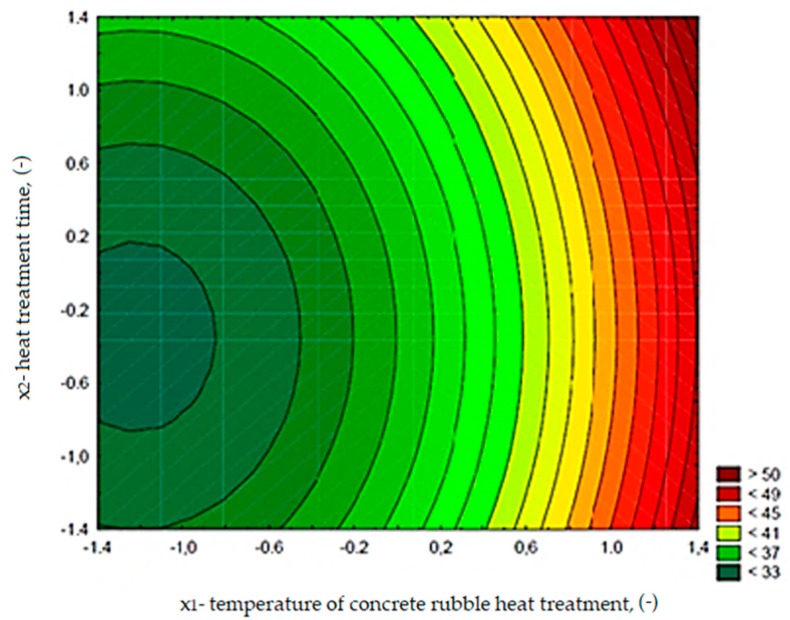
The changes in compressive strength of cement composites (MPa), depending on *x*_1_ and *x*_2_.

The changes in cement composite compressive strength depending on the calcination temperature of rubble (*x*_1_) and treatment time (*x*_2_) are presented in Figure 16. The function describing the dependence of the compressive strength on the tested variables for composites with RCM is expressed in the following equation:

(2) $$f_{cm,28}=36.16+5.48x_{1}+0.82x_{2}+2.24x_{1}^{2}+1.18x_{2}^{2}\;R^{2}=0.83$$

The obtained compressive strength test results indicate that the increase in calcination temperature from 288 to 712 °C caused the increase in compressive strength of about 32%. The highest strength values, exceeding those obtained for the control series, were recorded for composites with RCM calcined at temperature 650 °C (series 3 and 4, Figure 16). The compressive strength for these series exceeded the results obtained for the control series 11 by 4 and 7%, respectively. However, in case of using the RCM calcined at lower temperatures (up to 350 °C), as well as in the presence of non-calcined addition, the compressive strength results of the composite did not exceed 35 and 30 MPa, respectively. Thus, only a sufficiently high temperature of rubble processing allows to obtain RCM that causes a favorable effect on the compressive strength of cement composites.

#### 5.3.2. Flexural Strength

**Figure 17:**
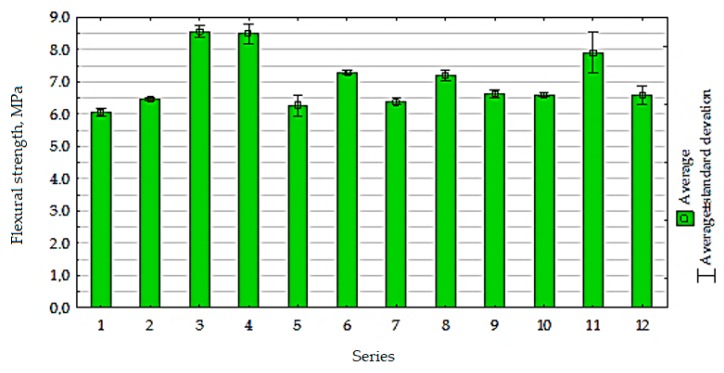
The flexural strength of composites with RCM.

The average flexural strength results obtained for the test series in comparison to the control samples (series 11) and (series 12) are given in Figure 17.

**Figure 18:**
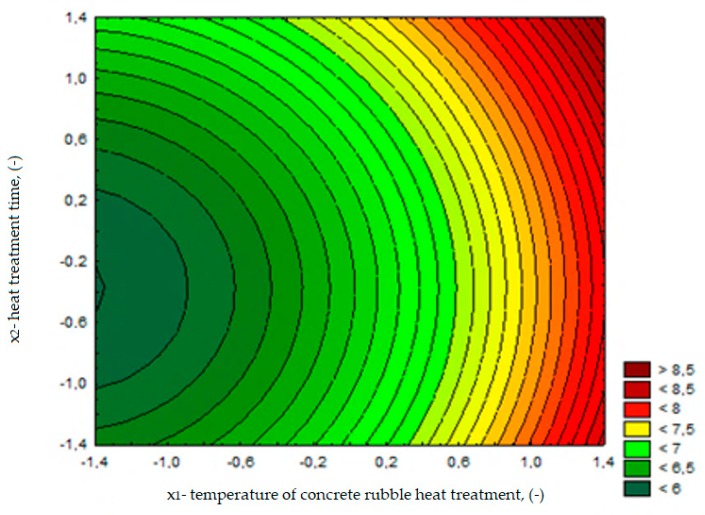
The changes in flexural strength of cement composites (MPa), depending on *x*_1_ and *x*_2_.

The changes in flexural strength depending on the calcination temperature of rubble (*x*_1_) and treatment time (*x*_2_) are presented in Figure 18. The function describing the dependence of the flexural strength on the tested variables for composites with RCM is expressed in the following equation:

(3) $$f_{fm,28}=6.76+0.75x_{1}+0.18x_{2}+0.24x_{1}^{2}+0.24x_{2}^{2}\;R^{2}=0.78$$

The analysis of test results showed that the most favorable results of flexural strength of cement composites containing RCM were obtained in cases of using the recycled material after calcination at temperature higher than 650 °C (series 3, 4, 6), and the results were similar to those obtained in the control series. The use of RCM from rubble after thermal treatment at lower temperatures (<650 °C) had no significant effect on flexural strength compared to the composite with non-calcined RCM.

#### 5.3.3. Water Absorbability

**Figure 19:**
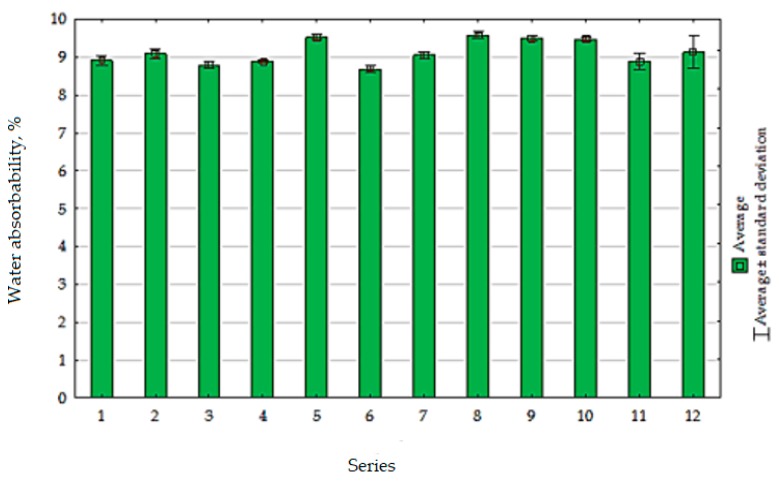
The water absorbability of composites with RCM.

The average results of water absorbability test were given in Figure 19.

**Figure 20:**
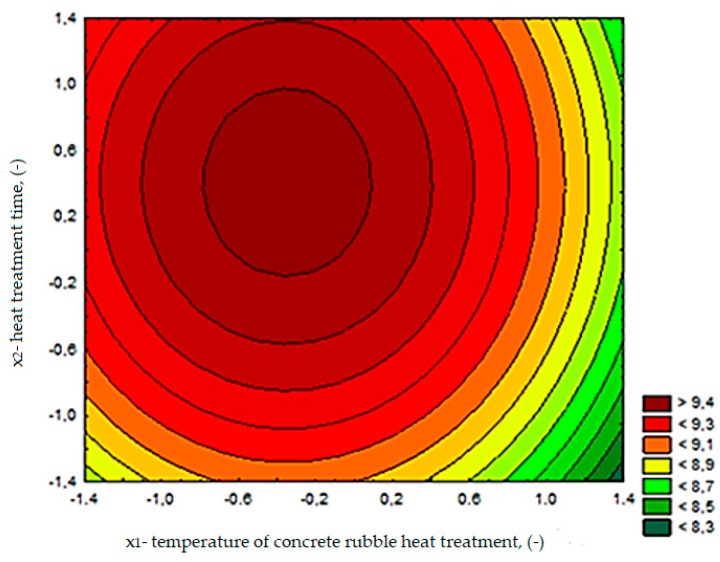
The water absorbability (%) changes of cement composites depending on *x*_1_ and *x*_2_.

The changes in cement composite water absorbability depending on the calcination temperature of rubble (*x*_1_) and thermal treatment time (*x*_2_) are presented in Figure 20. The function describing the dependence of water absorbability on the tested variables for composites with RCM is expressed in the following equation:

(4) $$WA=9.49-0.18x_{1}+0.13x_{2}-0.2x_{1}^{2}-0.16x_{2}^{2}\;R^{2}=0.68$$

Based on the test results, it can be concluded that a decrease in the absorbability of the cement composite occurs with the increase in RCM calcination temperature. In case of using RCM calcined at a temperature 650 °C or higher, water absorbability comparable to the absorbability of the control mortar was achieved. However, extended calcination time had a relatively negative influence on this property. It should be noted that the observed changes in water absorbability were relatively small and ranged from 8.7 to 9.6%. This is probably due to the fact that the RCM has a developed specific surface area similar to the cement used. The lowest water absorbability was recorded from the composite with RCM calcined at temperature 712 °C (series 6, Figure 20), which is also related to the test results of other parameters such as compressive strength or flexural strength.

## 6. Conclusions

Cementitious supplementary material used in cement composites was obtained as a result of thermal and mechanical treatment of concrete rubble as part of comprehensive recycling of reinforced concrete structures.

The statistical analysis of test results of compressive strength, flexural strength and water absorbability of mortars with RCM made it possible to determine the optimal conditions for production of cementitious supplementary material. It was found that the calcination temperature of concrete rubble had the most significant effect on the analyzed parameters of cement composites. The effect of calcination time was statistically less significant. The regression equations can be useful for estimation of the physical properties of composites with RCM considering the conditions of thermal treatment of concrete rubble.

The calcination of concrete rubble at a temperature of about 650 °C caused partial dehydration of cement hydration products, mainly the disintegration of portlandite (Ca(OH)_2_) into CaO and H_2_O. This treatment partially removed the hydration reactivity of old cement mortar, which resulted in improved physical properties of cement composites with RCM.

The results of extensive microstructural analysis, including X-ray diffractometry (XRD), differential thermal analysis (DTA), thermogravimetry analysis (TG) and scanning electron microscopy, confirmed the presence of non-hydrated cement, calcium hydroxide (CH), calcium oxide (CaO) and dicalcium silicate (C_2_S) in RCM, which are capable of hydration and creation of rehydration products. The influence of RCM treatment temperature on its rehydration reactivity properties was assessed based on the analysis of heat of hydration.

The proposed highly ecological solution for the management of waste generated in the concrete recycling process supports the idea of sustainable development by limiting the consumption of natural resources and reducing CO_2_ emissions generated during the cement production process. The test results showed that appropriate treatment of concrete rubble allows to obtain high-quality fine fraction which may be successfully used as a cement substitute or as pozzolanic additive for cement composites.

## Figures and Tables

**Table 1:** The composition of the initial concrete mix on 1 m^3^.

Component	Content
Cement CEM I 42.5 R, kg	360
Sand 0–2 mm, kg	641
Gravel 2–16 mm, kg	1170
Plasticizer, dm^3^	3.2
Water, dm^3^	144

**Table 2:** The composition of cement composites.

Component	Unit	Cement Composites	
		Series 1–10; 12	Series 11
Cement CEM I 42.5 R	g	337.5	450
Water	mL	225	225
Sand 0–2 mm	g	1350	1350
Recycled Cement Mortar	g	112.5	-

**Table 3:** Variables in the plan of experiment.

*X*_1_	Temperature of concrete rubble treatment, °C	288	350	500	650	712
*X*_2_	Thermal treatment time, min	30	40	60	80	90

**Table 4:** The rotatable central composite design of experiment.

Series	Real Values		Normalized Values	
	*X*_1_, °C	*X*_2_, min	*x*_1_	*x*_2_
1	350	40	−1	−1
2	350	80	−1	1
3	650	40	1	−1
4	650	80	1	1
5	288	60	−1.414	0
6	712	60	1.414	0
7	500	30	0	−1.414
8	500	90	0	1.414
9	500	60	0	0
10	500	60	0	0

**Table 5:** Content of selected components of RCM.

Type of RCM	Components of the RCM, % of Mass				
	Bound Water			Ca(OH)_2_	CaCO_3_
	H_I_	H_CH_	Σ		
Non-calcined	6.81	3.27	10.08	13.44	10.44
Calcined	-	0.24	0.24	0	25.79

**Table 6:** The flow diameter of cement composites mixes**.**

Type of Mixture	Flow Diameter, mm
Standard cement mortar (series 11)	170
Cement composite with RCM (series 4)	150
